# The effect of S100A6 on nuclear translocation of CacyBP/SIP in colon cancer cells

**DOI:** 10.1371/journal.pone.0192208

**Published:** 2018-03-13

**Authors:** Shanshan Feng, Qiaozhi Zhou, Bo Yang, Qianqian Li, Aiqin Liu, Yingying Zhao, Changqing Qiu, Jun Ge, Huihong Zhai

**Affiliations:** 1 Surgery Laboratory, General Hospital of Ningxia Medical University, Yinchuan, Ningxia Hui Autonomous Region, China; 2 Department of Gastroenterology, Beijing Friendship Hospital Affiliated to Capital Medical University, Beijing, China; 3 Beijing Key Laboratory for Precancerous Lesion of Digestive Diseases, Beijing, China; 4 National Clinical Research Center for Digestive Diseases, Beijing, China; University of Toronto, CANADA

## Abstract

**Background:**

Calcyclin Binding Protein/(Siah-1 interacting protein) (CacyBP/SIP) acts as an oncogene in colorectal cancer. The nuclear accumulation of CacyBP/SIP has been linked to the proliferation of cancer cells. It has been reported that intracellular Ca^2+^ induces the nuclear translocation of CacyBP/SIP. However, the molecular mechanism of CacyBP/SIP nuclear translocation has yet to be elucidated. The purpose of this study was to test whether the Ca^2+^-dependent binding partner S100 protein is involved in CacyBP/SIP nuclear translocation in colon cancer SW480 cells.

**Methods:**

The subcellular localization of endogenous CacyBP/SIP was observed following the stimulation of ionomycin or BAPTA/AM by immunofluorescence staining in SW480 cells. S100A6 small interfering RNAs (siRNA) were transfected into SW480 cells. Immunoprecipitation assays detected whether S100 protein is relevant to the nuclear translocation of CacyBP/SIP in response to changes in [Ca^2+^]*i*.

**Results:**

We observed that endogenous CacyBP/SIP is translocated from the cytosol to the nucleus following the elevation of [Ca^2+^]*i* by ionomycin in SW480 cells. Co-immunoprecipitation experiments showed that the interaction between S100A6 and CacyBP/SIP was increased simultaneously with elevated Ca^2+^. Knockdown of S100A6 abolished the Ca^2+^ effect on the subcellular translocation of CacyBP/SIP.

**Conclusion:**

Thus, we demonstrated that S100A6 is required for the Ca^2+^-dependent nuclear translocation of CacyBP/SIP in colon cancer SW480 cells.

## Introduction

CacyBP(Calcyclin-binding-protein) was initially shown in Ehrlich ascites tumor cells to interact with S100A6 at a physiological range of Ca^2+^ concentrations [1]. CacyBP is also called SIP (Siah-1 interacting protein) because a human ortholog of mouse CacyBP was shown to interact with Siah-1 [2]. CacyBP/SIP binds several target proteins such as several calcium binding proteins of the S100 family, Skp1, tubulin and ERK1/2. Through these protein-protein interactions, CacyBP/SIP plays important roles in cellular processes such as ubiquitination, proliferation, differentiation, tumorigenesis, cytoskeletal rearrangement and regulation of transcription [3–5].

Our group has generated a monoclonal antibody against CacyBP/SIP [6] and, using this specific antibody and immunohistochemistry, has shown that CacyBP/SIP is over-expressed in colon cancer tissue and is mainly localized in the nucleus [7]. However, the mechanism of accumulation of CacyBP/SIP in the nucleus of colon cancer cells is still not known. Previous studies in neuroblastoma cell lines have suggested that CacyBP/SIP is present in the cytoplasm and translocates to the perinuclear region or to the nucleus after elevation of intracellular Ca^2+^ concentrations [Ca^2+^]*i* [8,9]. Our group characterized a similar translocation of CacyBP/SIP from the cytosol to the nucleus in colon cancer cell lines [10] and gastric cancer cells [11], following treatment with gastrin, which also changed the [Ca^2+^]*i*. This led us to test whether elevated Ca^2+^ also causes the nuclear translocation of CacyBP/SIP. Among the multiple protein-binding partners of CacyBP/SIP, only S100 proteins bind to CacyBP/SIP in a Ca^2+^-dependent manner. In this study, we tested the hypothesis that S100 proteins are involved in the nuclear translocation of CacyBP/SIP following the elevation of intracellular Ca^2+^ concentration in colon cancer SW480 cells.

## Materials and methods

### Cell culture

Human colon cancer SW480 cells lines, which are primary CRC cell lines, were preserved in our laboratory. These cells have a high expression level of CacyBP/SIP [12]. SW480 cell lines were cultured at 37°C in a 5% CO_2_ incubator in DMEM (Gibco Invitrogen, Carlsbad, CA, USA) supplemented with 10% fetal bovine serum (FBS) (Gibco Invitrogen, Carlsbad, CA, USA), 100 U/ml of penicillin and 100 μg/ml of streptomycin.

### Loading the cells with ionomycin or BAPTA/AM

Colon cancer SW480 cells (2 x10^4^) were seeded onto coverslips in six-well plates and cultured for 2 days in DMEM. Cells were washed twice for 5 min in PBS and incubated with serum-free medium, 0.1% DMSO and an increasing amount of ionomycin (0, 1, 2, 5, 10 μmol/L) for 30 min at 37°C. Ionomycin (Merck-Millipore, Germany) was dissolved in DMSO (Gibco Invitrogen, Carlsbad, CA, USA) at different concentrations (0, 1, 2, 5, 10 μmol/L). Control cells were treated for 8–12 h with 0.1% DMSO. BAPTA (Santa Cruz Biotech, Santa Cruz, USA) was prepared as 5 μmol/L stock solutions (adjusted to pH 7.8 with NaOH) in anhydrous DMSO and stored at 4°C until use. Cells were treated in the same manner with 5 μmol/L of ionomycin plus different concentrations of BAPTA/AM (0, 5, 10, 25 μmol/L) for 1 hr at 37°C. After treatment, immunofluorescence staining was prepared for analysis using a META-510 Laser Scanning Confocal Imaging System (ZEISS, Germany).

### Immunofluorescence staining

The cells were grown on coverslips for 2 days in DMEM and treated with ionomycin and/or BAPTA/AM, fixed with 4% paraformaldehyde for 20 min at room temperature, washed with PBS, and permeabilized for 10 min with 0.3% Triton X-100 at 37°C. Then, cells were incubated with anti-CacyBP/SIP antibody (diluted 1:100 in PBS) overnight at 4°C after blocking with 5% normal goat serum for 30 min at room temperature. Finally, the cells were incubated with FITC-conjugated anti-mouse IgG (diluted 1:200) (Santa Cruz Biotech, USA) for 1h at room temperature. Nuclei were stained with DAPI for 5 min. Immunofluorescence was analyzed with a META-510 Laser Scanning Confocal Imaging System.

http://dx.doi.org/10.17504/protocols.io.mqcc5sw. [PROTOCOL DOI]

### Cytosolic free Ca^2+^ measurement

Cells grown on coverslips were placed on the homoeothermic platform of a confocal laser microscope for 12 h at 37°C and then treated with different concentrations of ionomycin for 30 min. The cells were washed with PBS three times and loaded with 20 μmol/L of Fluo-3/AM (Molecular Probes, AAT Bioquest, USA) dissolved in DMSO for 45 min at 37°C. Fluo-3/AM was excited at 506 nm, and the emitted fluorescence was filtered through a 526 nm filter, captured at a resolution of 512×480 pixels and digitized into 256 gray levels. Fluo-3/AM intensity was analyzed using the META-510 Laser Scanning Confocal Imaging System and the accompanying MultiGauge software. The fluorescence was monitored every 2 sec for 3 min. The fluorescence intensity was calculated by averaging all of the pixels in SW480 cells that were untreated and treated with ionomycin. The cells treated with 5 μmol/L of ionomycin plus different concentrations of BAPTA/AM (0, 5, 10, 25 μmol/L) were analyzed in the same way.

### Immunoprecipitation assays

Harvested cells treated with 5 μmol/L of ionomycin for 30 min were lysed by passing them through a 26 G needle 10 times in a buffer containing 1% SDS, 1 mmol/L of Na_3_VO_4_, 0.1 mol/L of Tris (pH 7.4), and protease inhibitors (10 mg/L of leupeptin, 5 mg/L of aprotinin, 20 mg/L of soybean trypsin inhibitor, and 1 mmol/L of phenylmethylsulfonyl fluoride). To distinguish cytosolic from nuclear CacyBP/SIP, cell fractions were extracted using the NE-PER™ nuclear and cytoplasmic extraction kit (Pierce Biotechnology, Rockford, IL). The extracts were centrifuged at 12,000 rpm for 25 min at 4°C. Proteins were quantified using the Enhanced BCA Protein Assay Kit (Beyotime, Wuhan, China).

For immunoprecipitation, lysates were precleared with 20 μL of protein A+G Agarose (P2012, Beyotime Biotechnology, Shanghai, China) beads and transferred to a fresh 1.5 mL tube. The supernatant was removed after centrifuging at 1,500 rpm for 30 sec at 4°C and washed three times with 500 μL of lysis buffer (P0013G, Beyotime Biotechnology, Shanghai, China). A total of 2 μg of anti-CacyBP/SIP (diluted 1:300) or 2 μg of IgG (normal mouse IgG) with fully resuspended protein A+G was added to 500 μg of protein and was incubated overnight at 4°C. Then, the bead complexes were resuspended with 500 μL of lysis buffer, and the supernatant was removed following centrifugation at 1000 rpm for 3 min at 4° C; the lysis buffer wash was repeated three times. The precipitate was re-suspended in 20 μL of 1x SDS denatured loading buffer for western blot analysis.

Lysates and immunoprecipitates were resolved on 12% SDS-polyacrylamide gel and transferred to polyvinylidene fluoride (PVDF) membranes (Millipore, Billerica, MA, USA). The membranes were blocked in Tris-buffered saline containing 0.2% Tween 20 (TBST) and either 5% nonfat dry milk or 3% BSA for blotting of tyrosine phosphorylation. After blocking, the membranes were incubated with primary antibody (S100A1 1:200, S100A6 1:200) (Santa Cruz Biotechnology, Santa Cruz, USA) at 4°C overnight. Following a TBST wash and incubation with horseradish peroxidase-conjugated secondary antibody (1:2000, Santa Cruz Biotechnology, USA), proteins were detected using enhanced chemiluminescence (ECL) (West Pico, Thermo Scientific, USA) and visualized using the LAS 4000 system. The ECL signal intensity in peptide competition experiments was measured using the LAS 4000 system and the accompanying MultiGauge software. All blots shown are representative of at least three experiments.

http://dx.doi.org/10.17504/protocols.io.mqdc5s6[PROTOCOL DOI]

### Small RNA interference (siRNA) and cell transfection

An siRNA of scrambled sequences was used as a negative control (NC, GenePharma Inc., Shanghai, China). Three siRNAs targeting the S100A6 coding sequences 120 to 138 (S100A6-siRNA1), 349 to 367 (S100A6-siRNA2) and 624 to 642 (S100A6-siRNA3) were designed and aligned to the human genome database in a BLAST search to ensure that the chosen sequences were not highly homologous with other genes. The detailed sequences were as follows: for oligo-1, sense: 5’-gcgaaugugcguuguguaatt-3’, and antisense: 5’-uuacacaacgcacauucgcuu-3’; for oligo-2, sense: 5’ -guggccaucuuccacaagutt-3, and antisense: 5-acuuguggaagauggccactt-3’; and for oligo-3, sense: 5’-ccucucugagucaaauccatt-3’, and anti-sense: 5’-uggauuugacucagagaggtt-3’. Briefly, 1×10^6^ cells were seeded into each well of 6-well plates and cultured for 48 h. Cells were transfected with 7.5 μmol/L of each siRNA for 24 hrs in Opti-MEM medium without antibiotics and 5% fetal calf serum using Lipo-fectamine 2000 (Invitrogen, Carlsbad, USA) according to the manufacturer's transfection protocol. The efficiency of siRNA was measured by western blot and quantitative reverse transcription polymerase chain reaction (qRT-PCR) analysis.

http://dx.doi.org/10.17504/protocols.io.mqec5te [PROTOCOL DOI]

### Quantitative real-time PCR (qRT-PCR)

Total RNA was extracted from cells by the Total RNA Kit I (OMEGA, USA), and cDNA was reverse-transcribed by the Reverse Transcription cDNA kit (TransGen Biotech, Beijing, China). Relative gene expression was determined using SYBR® Select Master Mix (Life Technologies, USA). Primer sequences were as follows: S100A6 forward: 5’-gggagggtgacaagcacac-3’, reverse: 5’-agcttcgagccaatggtgag-3’, and β-actin forward: 5’-cattaaggagaagctgtgct-3’, reverse: 5’-gttgaaggtagtttcgtgga-3’. β-actin was used for controls and normalization. Relative expression levels compared with control samples were calculated in each experiment using the ΔΔCt method.

http://dx.doi.org/10.17504/protocols.io.mqfc5tn [PROTOCOL DOI]

### Statistical analysis

Values represent the mean±s.d. from at least three independent experiments, and statistical analyses were carried out with GraphPad Prism 5 (GraphPad Software, La Jolla, CA). Differences between the experimental groups were determined using Student’s *t*-test and one-way analysis of variance. A value of P<0.05 was considered significant, * P < 0.05, ** P < 0.01, *** P < 0.001.

## Results

### Nuclear translocation of CacyBP/SIP in response to elevated [Ca^2+^]*i* by ionomycin in SW480 cells

**Fig 1:**
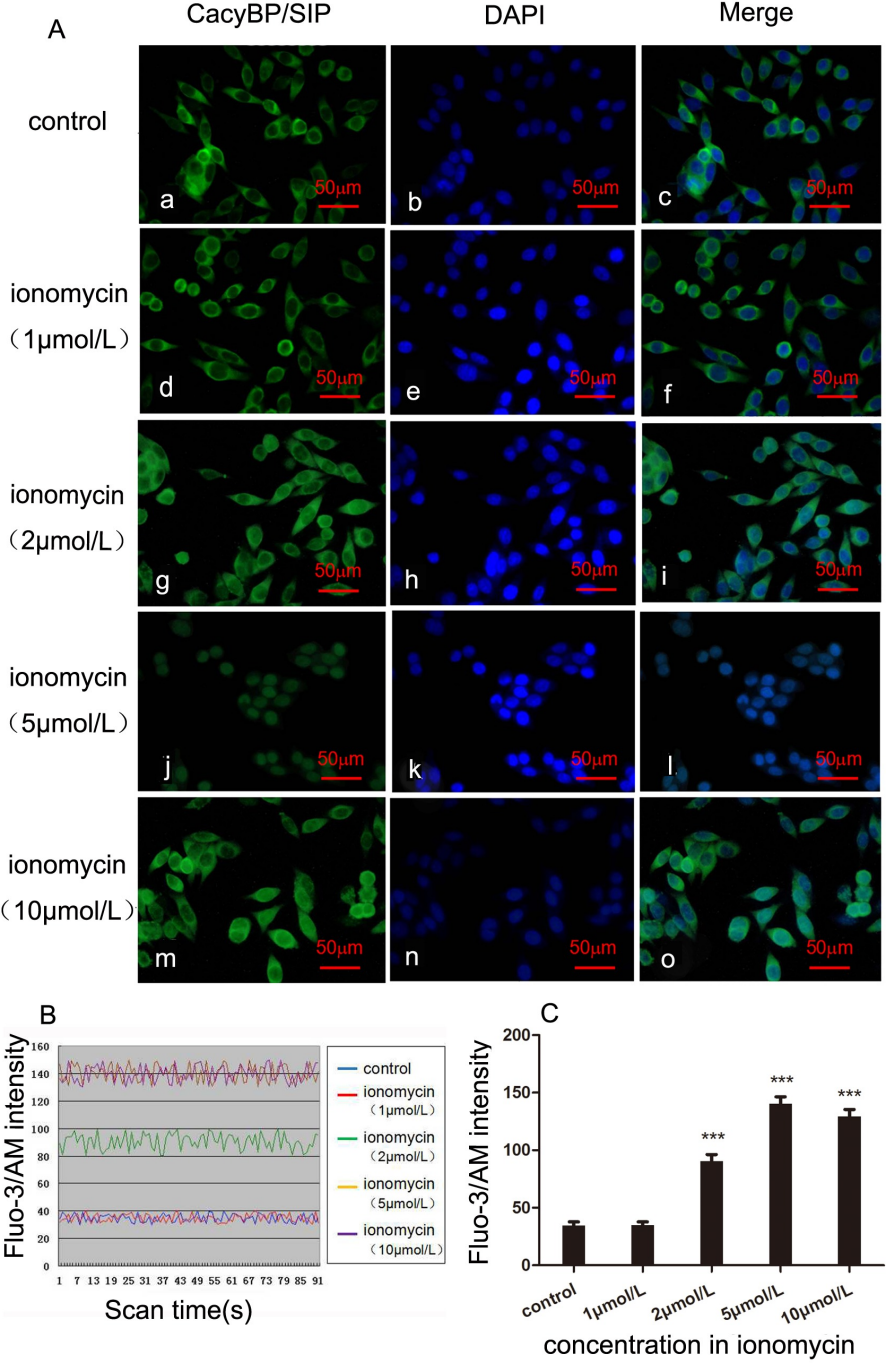
Effect of increased [Ca^2+^]i on the subcellular localization of CacyBP/SIP in colon cancer SW480 cells. (A) Effect of different concentrations of ionomycin on the localization of endogenous CacyBP/SIP. Cells were treated with ionomycin for 30 min, followed by immunostaining using anti-CacyBP/SIP, and were imaged with confocal microscopy. CacyBP/SIP was translocated to the perinuclear region in SW480 cells. After stimulation with an increasing amount of ionomycin (0, 1, 2, 5, 10 μmol/L) for 30 min at 37°C, SW480 cells were fixed and immunostained using CacyBP/SIP MAb (panels a, d, g, j, and m), and nuclei were labelled with DAPI (panels b, e, h, k, and n). The merged images are shown in panels c, f, i, l, and o. The scale bar represents 50 μm. (B) The intensity of cytosolic free intracellular Ca^2+^ fluorescence in SW480 cells treated with ionomycin (0, 1, 2, 5, 10 μmol/L). The Fluo-3 fluorescence intensity in SW480 cells reached a plateau at 5 μmol/L and 10 μmol/L of ionomycin. SW480 cells were loaded with 20 μmol/L of Fluo-3/AM for 45 min under a confocal microscope (495 nm). The fluorescence was captured every 2 sec and recorded for 3 min. (C) The bar chart shows the intracellular Fluo-3 intensity. Ca^2+^ concentration is increased by treatment with 2, 5, and 10 μmol/L of ionomycin (***P<0.*001*).

Preliminary results showed that colon cancer SW480 cells express more endogenous CacyBP/SIP protein than HT 29 cells and lovo cells [14]. Thus, the SW480 cells were chosen for further studies. To learn more about the effect of Ca^2+^ on the nuclear translocation of CacyBP/SIP, SW480 cells were treated with ionomycin at different concentrations to increase the intracellular Ca^2+^ concentration. The localization of CacyBP/SIP was compared in untreated and treated cells by immunocytochemistry staining using anti-CacyBP/SIP antibodies. In untreated SW480 cells, CacyBP/SIP was primarily distributed throughout the cytoplasm (Fig 1A). In ionomycin-treated cells, although CacyBP/SIP was still largely present in the cytoplasm when treated with 2 μmol/L of ionomycin (Fig 1A, (d, g)), and a small pool of CacyBP/SIP was present in the nuclei. Furthermore, 5 μmol/L of ionomycin caused a primarily nuclear localization of endogenous CacyBP/SIP (Fig 1A, j). However, 10 μmol/L of ionomycin induced CacyBP/SIP translocation less dramatically than 5 μM of ionomycin, and translocation more closely resembled that observed with 2 μM of ionomycin (Fig 1A, m).

The fluorescent calcium indicator Fluo-3/AM was used to measure the relative [Ca^2+^]*i*. The Fluo-3 fluorescence intensity of Ca^2+^ in ionomycin-stimulated SW480 cells (1, 2, 5, 10 μmol/L) was compared with that of untreated cells. The Fluo-3 fluorescence intensity in SW480 cells remained the same as untreated cells when treated with 1 μmol/L of ionomycin but increased when treated with 2 μmol/L of ionomycin and reached a plateau at 5 μmol/L and 10 μmol/L of ionomycin (Fig 1B and 1C), indicating that Ca^2+^ concentration is increased by treatment with ionomycin concentrations that are greater than 2 μmol/L (Fig 1C). We found that the Fluo-3 fluorescence intensity was slightly less in 10 μmol/L of ionomycin compared with 5 μmol/L (Fig 1C). We chose 5 μmol/L of ionomycin to stimulate SW480 cells for the following study. These findings suggest that an elevated intracellular Ca^2+^ concentration can change the subcellular localization of CacyBP/SIP in SW480 cells.

### BAPTA/AM abolishes the effect of ionomycin on the nuclear translocation of CacyBP/SIP

**Fig 2:**
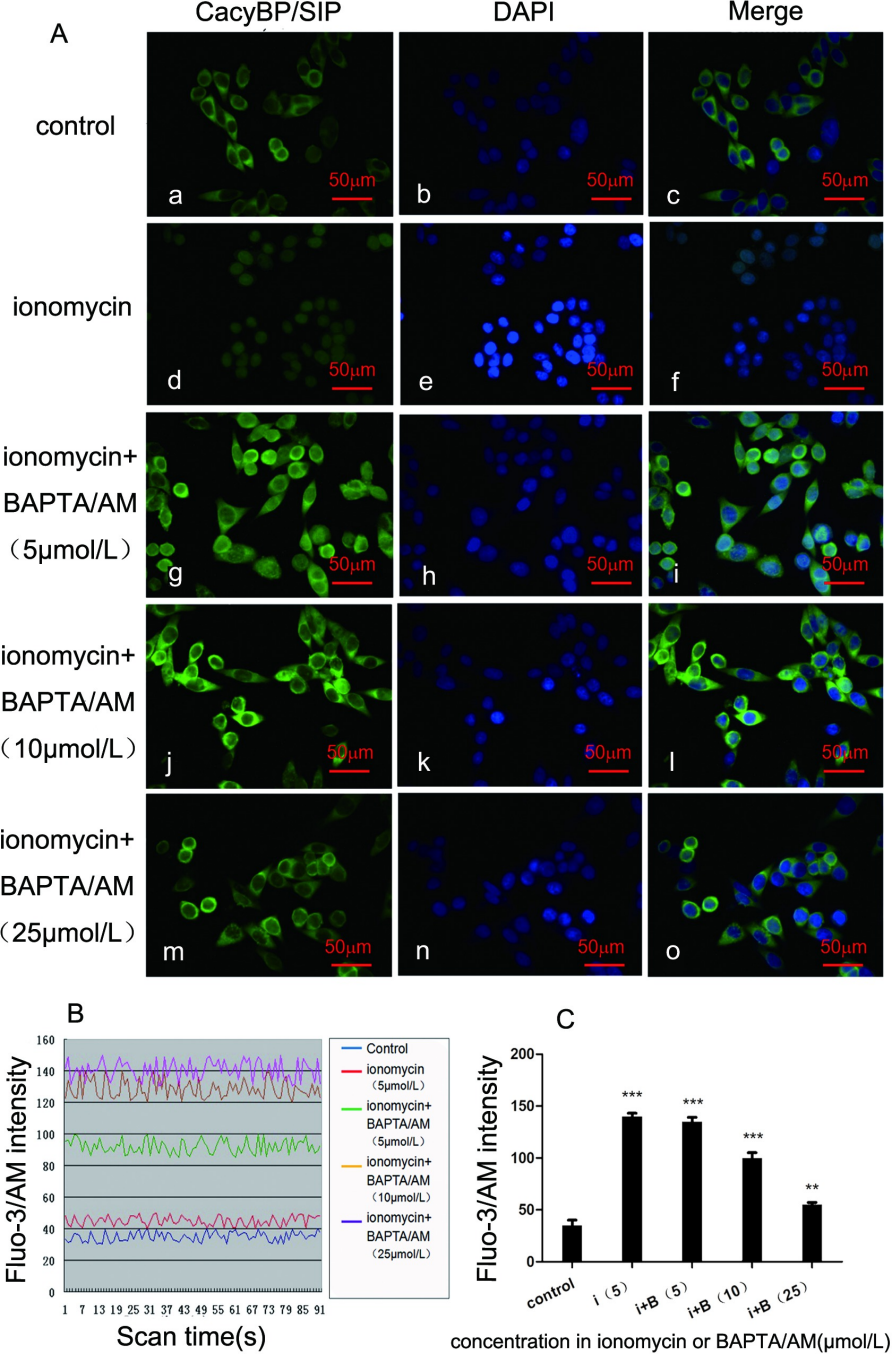
The effect BAPTA/AM on the nuclear localization of CacyBP/SIP induced by ionomycin in SW480 cells. (A) Effect of BAPTA/AM on ionomycin-stimulated nuclear translocation of endogenous CacyBP/SIP. Cells were treated with 5 μmol/L of ionomycin plus different concentrations of BAPTA/AM (0, 5, 10, and 25 μmol/L) for 30 min, followed by immunostaining using anti-CacyBP/SIP, and were imaged with confocal microscopy. Effect of different concentrations of ionomycin on the localization of endogenous CacyBP/SIP. SW480 cells were fixed and immunostained using CacyBP/SIP MAb (panels a, d, g, j, and m); nuclei were labeled with DAPI (panels b, e, h, k, and n). The merged images are shown in panels c, f, i, l, and o. The scale bar represents 50 μm. (B) The intensity of cytosolic free intracellular Ca^2+^ fluorescence stimulated by 5 μmol/L of ionomycin plus different concentrations of BAPTA/AM (0, 5, 10, and 25 μmol/L) in SW480 cells. BAPTA/AM at concentrations of 10 μmol/L and 25 μmol/L totally abolished the nuclear translocation of CacyBP/SIP induced by 5 μmol/L of ionomycin. Cells were loaded with 20 μmol/L of Fluo-3/AM for 45 min under a confocal microscope (495 nm). The fluorescence was captured every 2 sec and recorded for 3 min. (C) The bar chart shows the intracellular Fluo-3 intensity. The Fluo-3 fluorescence intensity was reduced by 5 μmol/L of ionomycin plus 10 μmol/L and 25 μmol/L of BAPTA/AM. Error bars represent the mean±s.d.

To confirm that the effect of ionomycin on the localization of CacyBP/SIP is dependent on intracellular [Ca^2+^]*i*, BAPTA/AM was used to monitor intracellular [Ca^2+^]*i*. Endogenous CacyBP/SIP was only slightly translocated from the nucleus to the cytoplasm when treated with both 5 μmol/L of ionomycin and 5 μmol/L of BAPTA/AM (Fig 2A). Moreover, 10 μmol/L and 25 μmol/L of BAPTA/AM totally abolished the nuclear translocation of CacyBP/SIP induced by 5 μmol/L of ionomycin (Fig 2B). In parallel, the Fluo-3 fluorescence intensity was also reduced by BAPTA/AM treatment (Fig 2C). Thus, we confirmed that the effect of ionomycin on the subcellular distribution of CacyBP/SIP is due to the change in intracellular [Ca^2+^]*i* in colon cancer SW480 cells.

### Interaction of CacyBP/SIP with S100A6 in a Ca^2+^-dependent manner in SW480 cells

**Fig 3:**
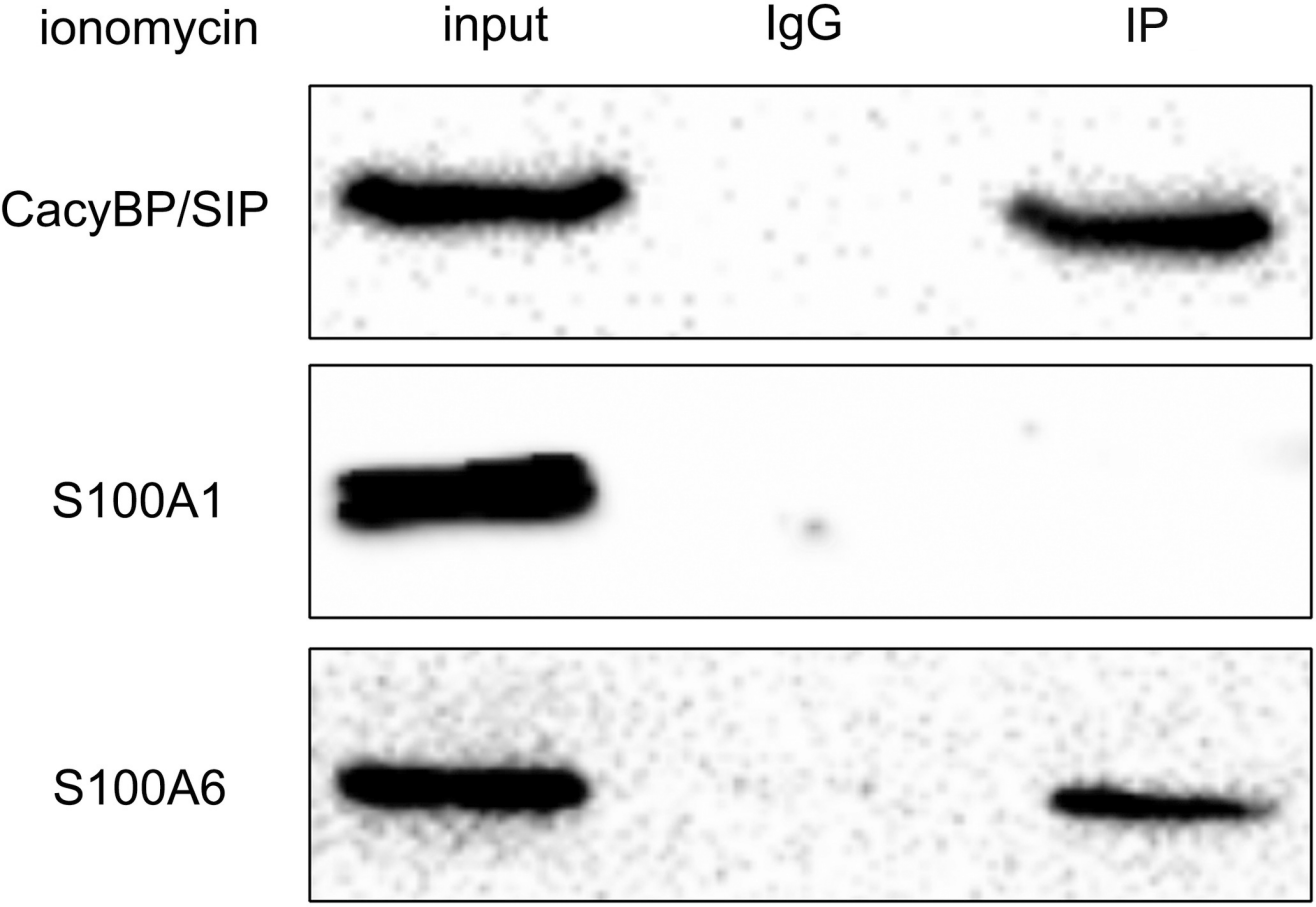
Interaction of CacyBP/SIP and S100A6 in a Ca2+-dependent manner in SW480 cells. CacyBP/SIP was precipitated by antibody against CacyBP/SIP, and the co-precipitation of S100A1 and S100A6 was analyzed by western blot analysis. The protein levels of CacyBP/SIP, S100A1, and S100A6 in cell lysates were analyzed by western blot analysis. Cells were treated with ionomycin (5 μmol/L) for 30 min. Data are representative immunoblots of three independent assays.

Among all the known protein-binding partners of CacyBP/SIP, only the binding of S100 proteins to CacyBP/SIP depends on Ca^2+^ concentrations [3]. The S100A1, S100A6, S100A12, S100B and S100P proteins all bind to CacyBP/SIP in a Ca^2+^-dependent manner [13]. It has been demonstrated that S100A12 is strongly expressed in inflammatory diseases [14]. S100B is mainly expressed in the brain and is strongly secreted by melanoma cells but is not expressed in colorectal cancer [14]. S100P is over-expressed in colorectal cancer but not in SW480 cells. Thus, S100A1 and S100A6 are candidates for our study to test whether their Ca^2+^-dependent interactions with CacyBP/SIP have effects on the nuclear translocation of CacyBP/SIP. Co-immunoprecipitation was used to examine the interaction of endogenous CacyBP/SIP with S100A1 and S100A6 in colon cancer SW480 cells. The nuclear CacyBP/SIP protein was extracted from 5-μmol/L ionomycin-treated SW480 cells. *Fig 3* shows that there was no interaction between S100A1 and CacyBP/SIP, whereas there was interaction between S100A6 and CacyBP/SIP. This suggests that S100A6 primarily interacts with CacyBP/SIP in a Ca^2+^-dependent manner (Fig 3).

### The effect of S100A6 on nuclear translocation of CacyBP/SIP

**Fig 4:**
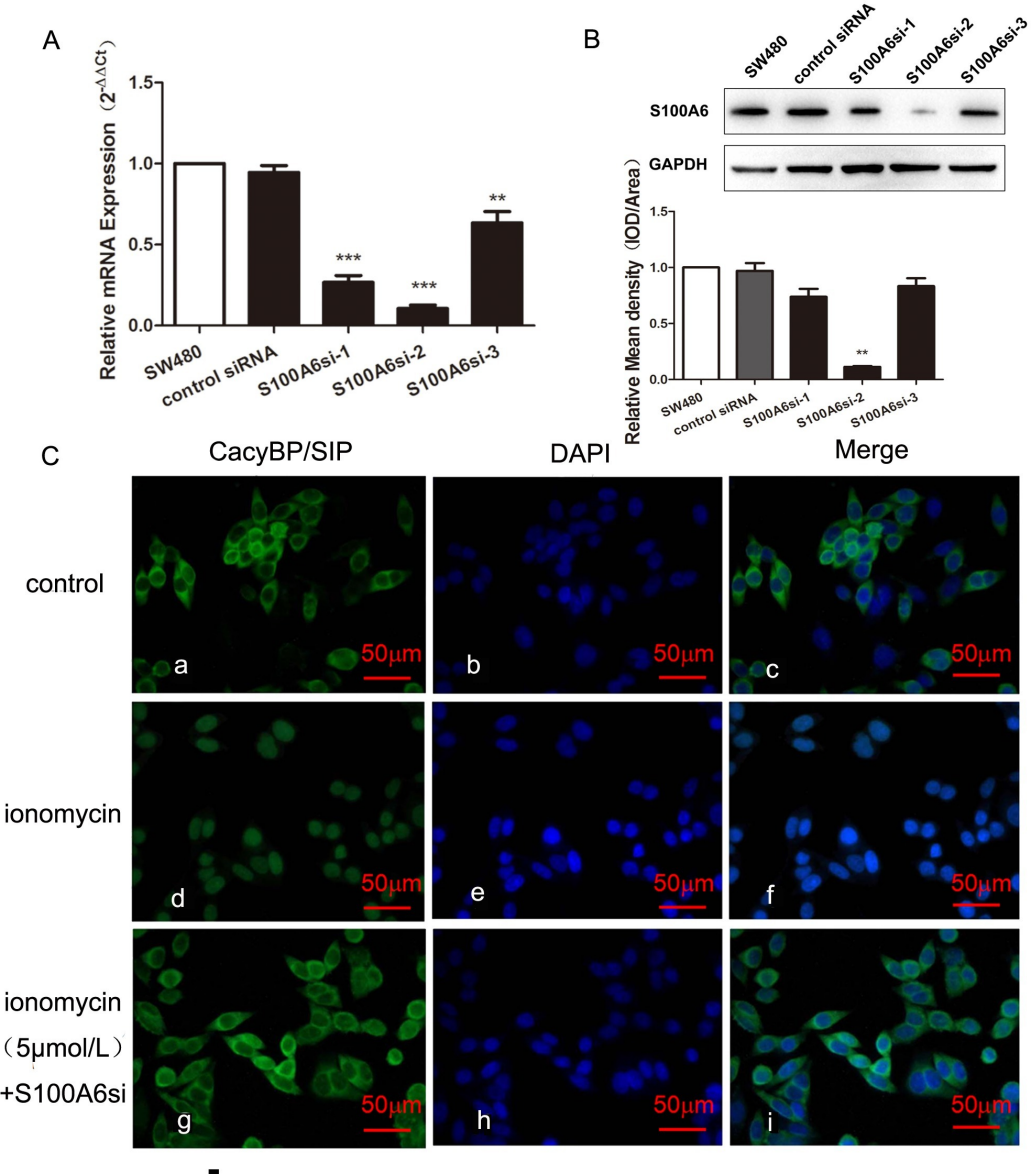
S100A6 is necessary for the Ca^2+^-dependent nuclear translocation of CacyBP/SIP. (A) The mRNA levels of S100A6 were measured by qRT-PCR. Total S100A6 mRNA in cells was examined by qRT-PCR. Student’s t-test, error bars represent the mean±s.d., **P<0.01, ***P<0.001, (S100A1si-1), (S100A1si-2) and (S100A1si-3) vs. SW480. (B) The protein levels of S100A6 were measured by western blot analysis. Data are representative immunoblots of three independent assays. The intensities were compared by one-way analysis of variance and Student’s t-test, error bars represent the mean±s.d., **P<0.01. (C) S100A6 knockdown abolished the Ca^2+^-dependent nuclear translocation of CacyBP/SIP. Cells were treated with 5 μmol/L of ionomycin plus S100A6si for 30 min, followed by immunostaining using anti-CacyBP/SIP, and were imaged with confocal microscopy. SW480 cells were fixed and immunostained using CacyBP/SIP MAb (panels a, d, and g), and nuclei were labeled with DAPI (panels b, e, and h). The merged images are shown in panels c, f, and i. The scale bar represents 50 μm.

To confirm whether S100A6 is involved in the Ca^2+^-dependent subcellular localization of CacyBP/SIP, S100A6 short-interfering RNA (siRNA) was employed to down-regulate the S100A6 transfectants. As shown in *Fig 4A* and *4B*, 2 siRNAs efficiently down-regulated CacyBP/SIP in SW480 cells at both the protein and mRNA levels by 85% and 87.9%, respectively. *Fig 4C* shows that CacyBP/SIP translocation was absent in response to treatment with 5 μmol/L of ionomycin when S100A6 was down-regulated by siRNA.

## Discussion

In this study, we demonstrated that in colon cancer SW480 cells, CacyBP/SIP was translocated from the cytoplasm to the nuclei in response to elevated intracellular Ca^2+^ by ionomycin. And we confirmed that CacyBP/SIP was also re-translocated from the nuclei to the cytoplasm by decreasing the intracellular [Ca^2+^]*i* with BAPTA in colon cancer SW480 cells. Furthermore, we found that the nuclear translocation of CacyBP/SIP was less at 10 μmol/L of ionomycin than at 5 μmol/L. Taken together, these data verified that intracellular [Ca^2+^]*i* plays a role in the localization of CacyBP/SIP in colon SW480 cancer cells.

A nuclear localization signal (NLS), which may induce CacyBP/SIP nuclear translocation, was identified in amino acids 143–159, based on the protein sequence analysis of CacyBP/SIP [9]. However, in the study by Ning, the eukaryotic expression vectors of wild-type CacyBP (FLAG-CacyBP) and a truncated mutant lacking the NLS domain (FLAG-CacyBPΔNLS) were constructed and transfected into MKN45 gastric cancer cells. Both flag-CacyBP and flag-CacyBPΔNLS were translocated to the nucleus by treatment with 5 μmol/L of ionomycin, indicating that Ca^2+^ is relevant to the subcellular localization but not to the NLS of CacyBP/SIP in gastric cells [13]. Regardless of these observations, CacyBP/SIP is found primarily in the cytoplasm under basal conditions. When cytosolic Ca^2+^ is elevated, CacyBP/SIP translocates from the cytoplasm to the nucleus [8, 9]. In this study, we also demonstrated that CacyBP/SIP is translocated from the cytoplasm to the nuclei in response to elevated intracellular Ca^2+^ by ionomycin in colon cancer SW480 cells and is re-translocated from the nuclei to the cytoplasm by decreasing the intracellular [Ca^2+^]*i* with BAPTA. Furthermore, we found that the nuclear translocation of CacyBP/SIP was observed less at 10 μmol/L of ionomycin than at 5 μmol/L of ionomycin. This supports the hypothesis that the intracellular calcium concentration reaches a transient peak in different cellular events, such as genetic transcription, cell cycle and protein synthesis [14]. Therefore, increasing the Ca^2+^ concentration plays a role in the nuclear translocation of CacyBP/SIP.

The S100 protein is the only Ca^2+^-binding target protein among the CacyBP/SIP binding partners such as Siah-1, Skp1, tubulin and ERK1/2 [3]. CacyBP/SIP was discovered as an S100A6 ligand at a physiological range of Ca^2+^ concentrations [1,2]. S100A1, S100A12, S100B and S100P were then shown to bind with CacyBP/SIP [11]. It has been demonstrated that residues 189–219 are involved in interactions with residues 155–229 of S100A6 [15]. It is tempting to speculate that S100A6 is relevant to the subcellular localization of CacyBP/SIP depending on Ca^2+^ concentrations. In our study, we found increased binding of S100A6 to CacyBP/SIP following the elevation of [Ca^2+^]*i* by ionomycin. S100A6 siRNA down-regulated the expression of S100A6 and diminished the ionomycin-stimulated nuclear translocation of CacyBP/SIP in SW480 cells. This indicates that S100A6 plays a critical role in the Ca^2+^-dependent nuclear translocation of CacyBP/SIP.

Indeed, previous studies reported that S100A6 could translocate between the cytoplasm and the nucleus in a calcium-dependent manner [12]. S100A6 is a low molecular weight Ca^2+^-binding protein (MW = 10.5 kD). S100A6 contains two EF-hand motifs responsible for the binding of Ca^2+^. Binding of Ca^2+^ induces a conformational change in the S100A6 molecule and in turn allows for interaction with target proteins and transduction of Ca^2+^ signals [16]. In addition, S100A6 has been implicated in a variety of cellular functions, including cell proliferation and tumorigenesis [17]. The increased interaction between S100A6 and CacyBP/SIP and their co-translocation from cytoplasm to the nucleus in response to elevated Ca^2+^ suggest that CacyBP/SIP and S100A6 cooperate with each other in regulating tumorigenesis. CacyBP/SIP is a component of the ubiquitin degradation pathway. CacyBP/SIP interacts with Siah-1 and Skp1 and is involved in the polyubiquitination and degradation of β-catenin, which is an oncogene associated with the Wnt signaling pathway [2,3]. Thus, the effect of S100A6 modulation of CacyBP/SIP translocation on the function of tumorigenesis, especially in colorectal cancer, is worthy of future studies.

In summary, we showed that S100A6 is involved in the nuclear translocation of CacyBP/SIP in a Ca^2+^-dependent manner in colon cancer SW480 cells. However, the detailed mechanism and functional significance of the nuclear translocation of CacyBP/SIP require further investigation.

## Supporting information

S1 Table The intracellular Fluo-3 intensity in SW480 cells treated with 2, 5, and 10 μmol/L of ionomycin. The table shows the intracellular Fluo-3 intensity. Ca^2+^ concentration is increased by treatment with 2, 5, and 10 μmol/L of ionomycin.
(XLSX)

S2 Table The intracellular Fluo-3 intensity in SW480 cells 5 μmol/L of ionomycin plus 10 μmol/L and 25 μmol/L of BAPTA/AM. The Fluo-3 fluorescence intensity was reduced by 5 μmol/L of ionomycin plus 10 μmol/L and 25 μmol/L of BAPTA/AM.
(XLSX)

S3 Table The relative protein mean density (IOD/Area) of CacyBP/SIP, S100A1 and S100A6. The protein levels of CacyBP/SIP, S100A1, and S100A6 in cell lysates. Data are representative immunoblots of three independent assays.
(XLSX)

S4 Table The mRNA levels of S100A6. The Relative mRNA Expression(2-ΔΔCt) levels of S100A6 were measured by qRT-PCR.
(XLSX)

S5 Table Relative protein mean density (IOD/Area) of S100A6. Total S100A6 mRNA in cells was examined by qRT-PCR. The protein levels of S100A6. Data are representative immunoblots of three in depend.
(XLS)

S1 Fig The protein level of CacyBP/SIP by western blot. (TIF)

S2 Fig The protein level of S100A1 by western blot. (TIF)

S3 Fig The protein level of S100A6 by western blot. (TIF)

S4 Fig The relative mRNA expression of GAPDH by qRT-PCR. (TIF)

S5 Fig The relative mRNA expression of S100A6si by qRT-PCR. (TIF)
