# Supplementary figures and images for: The effect of S100A6 on nuclear translocation of CacyBP/SIP in colon cancer cells
Source: PLoS One. 2018 Mar 13;13(3):e0192208. doi: 10.1371/journal.pone.0192208 (PMC5849316; doi:10.1371/journal.pone.0192208)

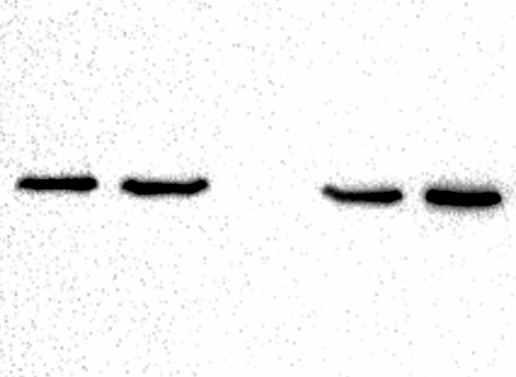

Supplement: S1 Fig — (TIF) [file pone.0192208.s006.tif]

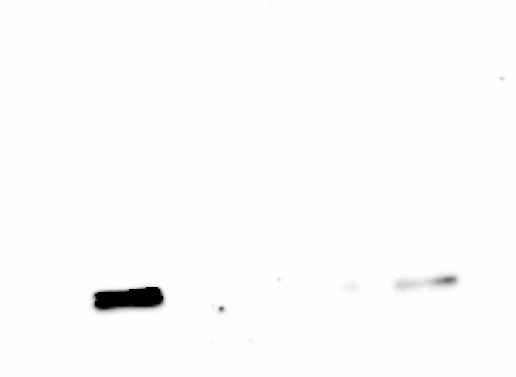

Supplement: S2 Fig — (TIF) [file pone.0192208.s007.tif]

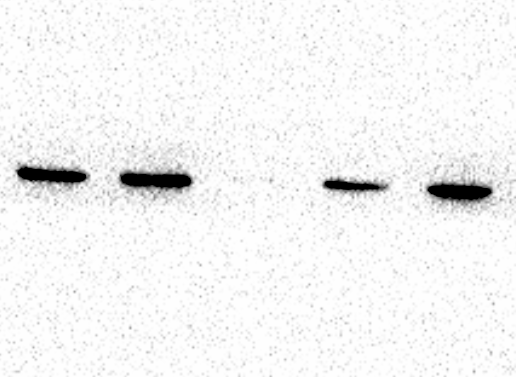

Supplement: S3 Fig — (TIF) [file pone.0192208.s008.tif]

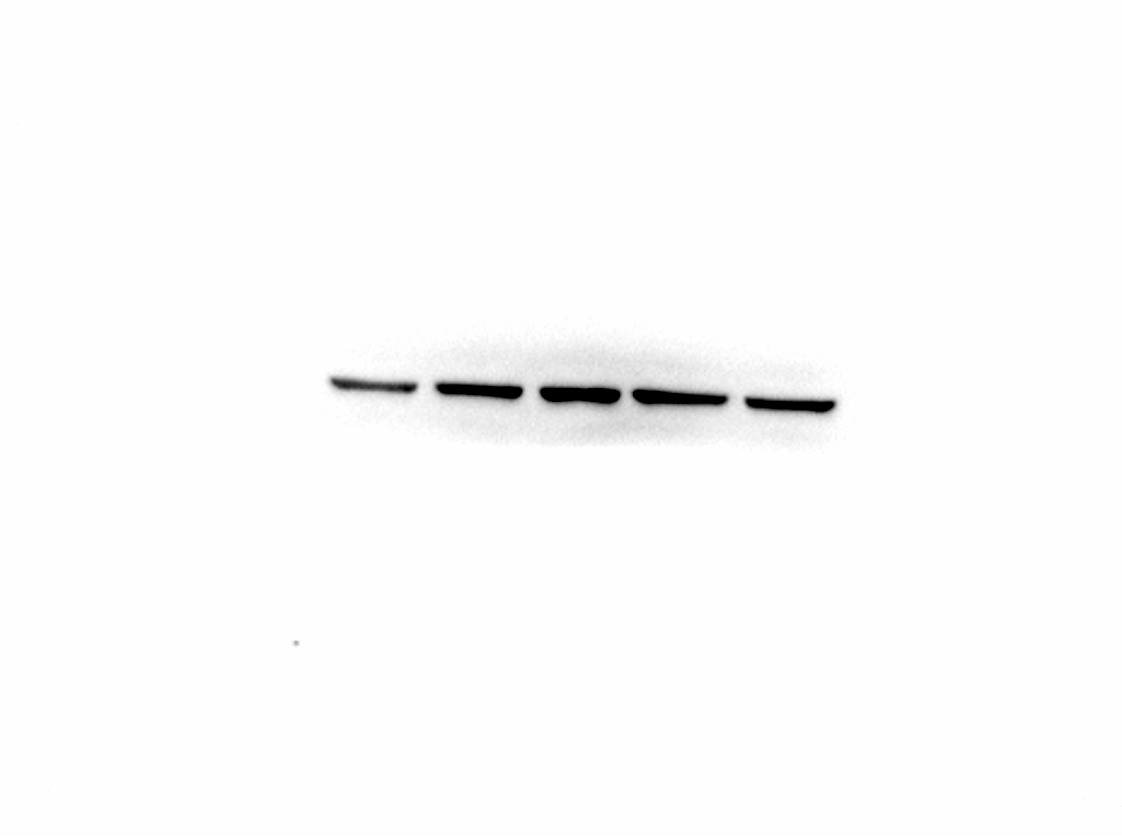

Supplement: S4 Fig — (TIF) [file pone.0192208.s009.tif]

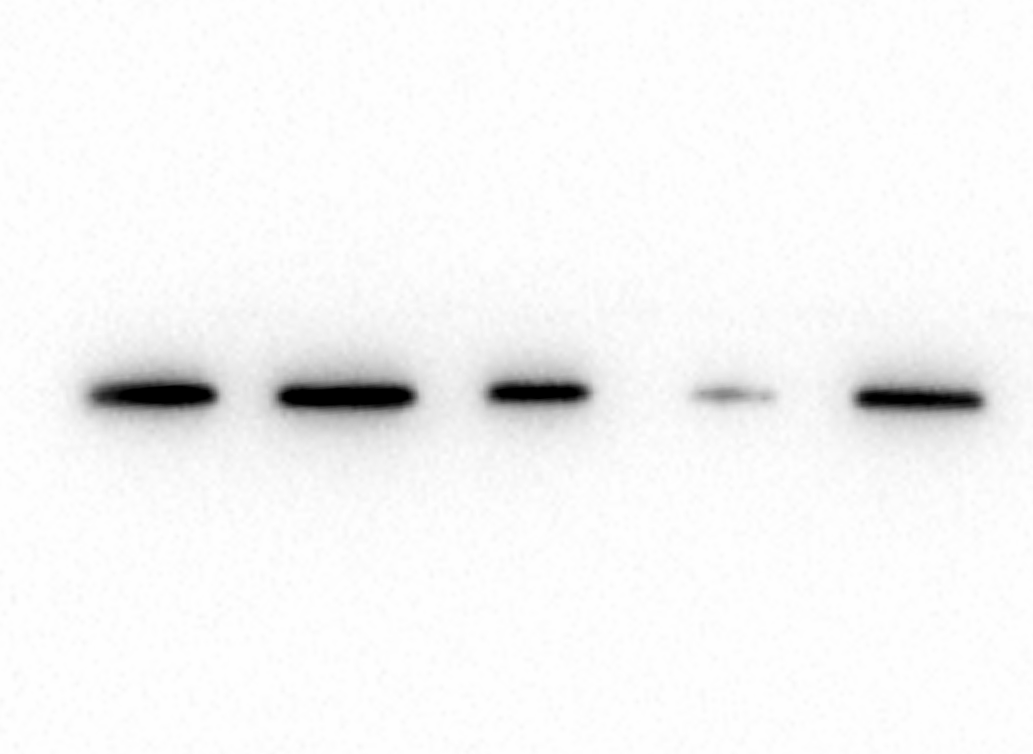

Supplement: S5 Fig — (TIF) [file pone.0192208.s010.tif]
